# Efficacy of an inflatable deterrent for reducing New World vulture human-wildlife conflict

**DOI:** 10.1038/s41598-024-56941-2

**Published:** 2024-03-19

**Authors:** Bryan M. Kluever, Betsy A. Evans, Noah M. Osterhoudt, Eric A. Tillman

**Affiliations:** United States Department of Agriculture, Wildlife Services, National Wildlife Research Center, Florida Field Station, Gainesville, FL 32641 USA

**Keywords:** Behavioural ecology, Animal behaviour, Conservation biology

## Abstract

Increasing urbanization coupled with spatial expansion and numerical increase of New World vulture populations has engendered a rise in human-vulture conflict, creating a need for effective tools to mitigate vulture-related damage. Visual frightening devices that mimic the presence of human or other predators can be employed in human-vulture conflict scenarios to increase perceived risk by the pest species, thereby eliciting an antipredator behavioral response, such as fleeing. One visual frightening device, inflatable scarecrows, recently proved effective at reducing passerine attendance at feral swine feeders, but their effectiveness when directed at other species and conflict scenarios has varied. Our primary objective was to evaluate an inflatable deterrent for reducing the number of black (*Coragyps atratus*) and turkey vultures (*Cathartes aura*) present (hereafter abundance) at 13 human-vulture conflict sites throughout the southeastern United States. We predicted that vulture abundance would be substantially reduced when inflatable deterrents were deployed. Because we suspected other factors might also influence vulture site abundance, we also examined the exploratory variables of weather, site size (area), and vulture tolerance to human approach in relation to vulture site abundance using a model selection approach. Black vulture site abundance was more pervasive than turkey vultures, occurring at all sites and accounting for 85% of daily vulture counts (10.78 ± 0.52 vultures/site/day) whereas turkey vultures were only present at 62% of sites (2.12 ± 0.21). Across all sites, inflatable scarecrows were effective at reducing vulture abundance by 82% during the seventeen-day treatment period when deterrents were deployed (3.50 ± 0.20), but only a 48% reduction during the twenty-one-day post-treatment phase (15.34 ± 1.39) was observed. Site size and weather did not influence tool effectiveness. Human tolerance at sites, as determined by vulture flight initiation distance, was influential, with tool effectiveness being reduced at sites where local human tolerance was high. We recommend inflatable scarecrows as a tool to reduce vulture-wildlife conflict to private property and recreation at sites where the conflict is spatially restricted (e.g., parking lot or recreation area), conducive to scarecrow deployment (e.g., flat stable surfaces), and where vulture site human tolerance is low to moderate.

## Introduction

Globally, many vulture species (Accipitridae and Cathartidae) have recently experienced population and/or distribution declines^1–3^. Contrarily, over the past several decades, North American populations of black (*Coragyps atratus*) and turkey vultures (*Cathartes aura*) have increased in abundance and expanded their distribution^4–6^. Concomitantly, there has been an increase in human-vulture conflicts involving these species, particularly in the eastern and southern United States^7–9^.

Large congregations of both black and turkey vultures at roosting, loafing, or foraging sites can be associated with human-vulture conflict^4,7^. Conflicts associated with black vultures include damage to residential and commercial property^8^, such as the destruction of rubber and vinyl type (hereafter termed synthetic) products (e.g., roof coverings, vinyl seat covers, windshield wipers), depredation of livestock^10,11^, and potential risks to human health and safety through exposure to fecal matter and aircraft collisions^6^. Conflicts with turkey vultures are similar to those experienced with black vultures with the exception that the species is less predatory and does not represent a livestock depredation threat^10^.

Due to considerable economic losses incurred as a result of vulture-related conflicts^4,9^ a variety of mitigation techniques have been employed in an attempt to minimize damage^10^. These methods have primarily focused on dispersal of roost sites^12,13^, reducing aircraft collision risks^14^, and the creation of allowable take models^6^ that can allow for and justify lethal removal. In addition, wildlife managers and others experiencing vulture damage have utilized deterrents such as lasers, vulture effigies, motion-activated sprinklers, and inflatables in an attempt to reduce vulture conflicts at residential and commercial properties^10^. The latter tool’s intended effectiveness and underlying driving mechanism may be rooted in antipredator behavior and theory^15^ and is intended to leverage the fear or perceived risk exhibited by the species of interest^16^. However, an organism’s fear/perceived risk of objects can be attributable to their novelty rather than the perception of objects or cues as a potential predator, and both can elicit similar behavioral responses^17,18^. For vultures, our understanding of antipredator and/or novel object behavioral responses within a human-wildlife context is lacking. For example, Pfeiffer et al.^19^ examined turkey vulture responses to various UAS platforms and approaches and found that the more “predator looking” platform did not elicit a greater behavioral response. For black and turkey vultures, aversive behavioral response to effigies has been well documented^20,21^, but whether this behavior is driven by an antipredator, novel object, or alternative response remains unknown.

Often, reports of the effectiveness of tools for reducing vulture-human conflict are anecdotal, based on studies with limited statistical inference due to small sample sizes, and/or only incorporate a singular study site into their study design^10^. For the latter, inference to populations of vultures not associated with singular study sites is limited but nonetheless occurs^10^. In the absence of the ability to employ random sampling at the study site spatial extent, which is often the case in wildlife field studies, incorporating multiple rather than singular study sites is recommended^22^. As such, evaluation of tools intended to reduce vulture-human conflict that include multiple study sites are needed to better inform wildlife managers and the general public^9^.

Inflatable scarecrows, an automated visual deterrent, have been tested for reducing human-wildlife conflict for several species, with mixed results. At blueberry farms and grape vineyards, fruit depredating passerines were not highly deterred by inflatable tube men (LookOurWay®, San Francisco, CA USA)^23,24^. For fish depredating double-crested cormorants (*Phalacrocorax auritus*) at aquaculture ponds, the “Scarey Man® Fall Guy” (R. Royal, Midnight, MS, USA) initially reduced bird attendance but effectiveness declined after one to two weeks of tool deployment^25,26^. Similarly, deployment of an inflatable deterrent initially resulted in only 25% of dingoes (*Canis dingo*), accessing food, but after only three trials 42% accessed food^27^. Most recently, Snow et al.^28^ found that the scare dancer (Snake 6 ft Cordless Inflatable Scarecrow, AirCrow LLC, Lake Charles, LA, USA) reduced bird visitation at wild pig feeding stations by 96 to 100%; bait stations were ephemeral, potentially negating the likelihood of habituation occurring. In a literature review focused on small, invasive pest birds, Klug et al.^29^ identified 32 investigations testing visual deterrents for reducing wildlife damage. Reported advantages included portability, initial affordability, and inexpensive operation, whereas disadvantages included habituation, limited range, and need to routinely move deterrents.

Several trends regarding the above studies and others investigating deterrent effectiveness are evident. First, replication in terms of study sites/sampling units is usually singular or sparse. Second, when multiple sites are incorporated into study design they are not evaluated/compared for site specific differences. And third, habituation by target species/animals reportedly reduced deterrent effectiveness in most cases.

Our overall objective was to determine the efficacy of an inflatable deterrent device for reducing the number of vultures present at localities reported as human-vulture conflict areas. We predicted that (1) the inflatable deterrent device would appreciably reduce vulture abundance across sites, (2) vulture abundance would increase with increased distance from the inflatable deterrent, (3) that deterrent effectiveness would differ across sites and (4) deactivation of inflatable deterrent devices would lead to an increase in vultures.

## Methods

### Study area and timing

**Figure 1:**
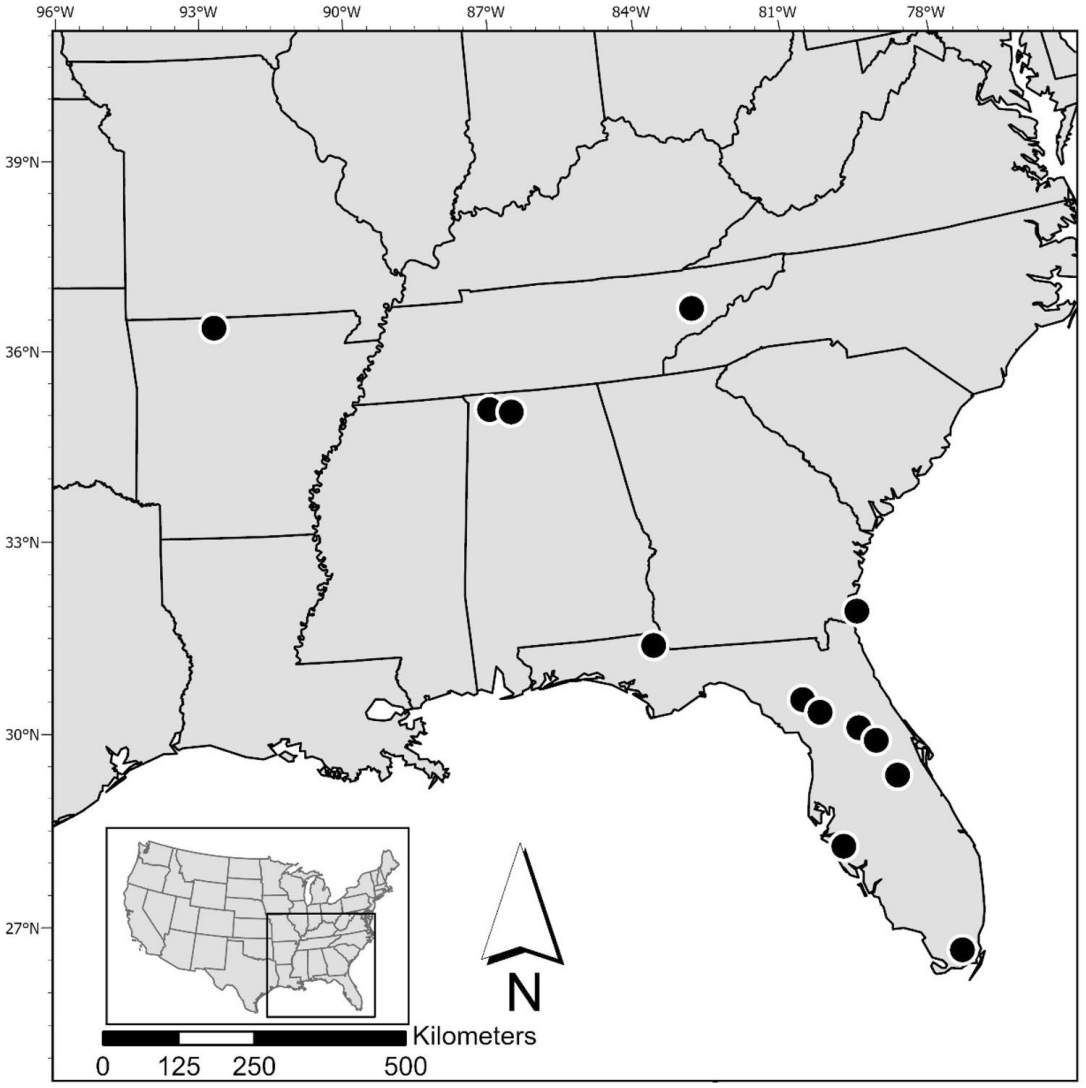
Map of study site locations (*n* = 13), southeastern United States, 2021–2023. The map projection is Lambert Conformal Conic.

Our study area included 13 vulture-human conflict sites across the southeastern United States that spanned five states (Fig. 1). Sites varied by type (i.e. parking lots, hydroelectric dams, baseball fields, small-scale farms, waste management sites, etc.), but all had current occurrences of vulture-related conflict. These sites were selected through consultation with U. S. Department of Agriculture Wildlife Services Operational Program State Directors and subsequent site visits with managers of individual sites. Twenty sites were proposed as potential study sites; however, seven sites were removed from consideration due to a lack of consistency in vulture presence necessary to complete each phase of the study. All of the sites had a history of vulture-related property damage (Table S1).

We conducted study trials year-round, beginning September 2021 and ending July 2023 (Table S1). Both black and turkey vultures are known to be year-round residents in the southeastern United States where our trials were conducted. North American populations of turkey vultures are known to migrate from the northern part of their range south during the wintertime, with large numbers of birds making their way to the southeastern United States^4^. Black vultures are not considered migratory in the southeastern United States and are broadly defined as central place foragers^30^. Hence, black vulture movements in our study area were more likely more local in nature^31^. During the course of our study, trials conducted during turkey vulture migration season (generally mid-September through mid-April for our study area) may have encountered both resident and migratory individuals, whereas trials conducted outside of migration season were likely to encounter resident birds only.

### Inflatable deterrent

We selected the Scare Dancer® (Air Crow® LLC, Lake Charles, LA, USA) as our inflatable deterrent for testing due to its portable size, ability to be battery powered, options for intermittent operation via motion sensor or timer, and its reported effectiveness for repelling passerine species^28^. The Scare Dancer® was equipped with a 1.83 m inflatable tube and mounted approximately 1 m off the ground on a t-post. The unit was powered using a 12 V 12AH Sealed Lead Acid (SLA) rechargeable battery. We initially equipped each unit with a motion sensor; however, in preliminary tests with captive vultures, we found the sensors were not reliable in detecting vulture movement. Therefore we equipped each unit with a timer to introduce intermittent action. After consulting with the manufacturer regarding battery life of the units, we set each timer to activate for 1 min every 5 min for 2-h. We selected a 2-h window due to the limitations of the batteries, but also because vultures used most sites in the afternoon or morning as pre- or post-roost sites and this window could be timed so that unit was deployed during periods of greatest activity. Units were deployed once per day, within 30 min of the start time from the first day of unit deployment. Periods of operation for units varied between sites as each site was unique in time of peak vulture use, as determined during the pre-treatment phase.

### Study design

To evaluate the efficacy of the inflatable deterrents for reducing vulture abundance, we tested the deterrent in three consecutive phases (Table 1) at selected study sites: pretreatment (inflatable deterrents not activated), treatment (inflatable deterrents activated), and post-treatment (inflatable deterrents not activated). We determined time of data collection based on when vultures were reliably present at each site. At a given site, we collected data within 30 min of the same time each day. During all three study phases, inflatable deterrent equipment was placed prior to the arrival of vultures at the site to ensure that observer presence and installation did not influence vulture behavior. At each site we placed four inflatable deterrent units. Units were placed between 10 and 20 m apart depending on site characteristics and in areas where vulture damage occurred most frequently. During the treatment phase, the units were activated according to the schedule described above.

**Table 1:** Study design used to test the effectiveness of inflatable deterrents at sites (*n* = 13) across the southeastern United States, 2021–2023.

Phase	Time period	Data to be collected
Pretreatment (Inflatable deterrent not activated)	3 days	2 h observation period with vulture counts collected every 15 min
Treatment (Inflatable deterrent activated)	3 consecutive days + 2 weeks (1 observation/week) to evaluate potential habituation	2 h observation period with vulture counts collected every 5 min
Post-treatment (Inflatable deterrent not activated)	3 weeks (1 observation/week)	2 h observation period with vulture counts collected every 15 min

The number of vultures present (hereafter vulture abundance), species composition (black and/or turkey vultures), and age class (hatch-year or after hatch-year) were recorded at 15-min intervals during each two-hour observation period. During the treatment phase, the distance of vultures from the deterrents was recorded using binned categories (1–10 m, 11–25 m, 26–50 m, 50 + m) in order to determine the range of effectiveness of the stimuli.

Observations of vultures during initial site visits anecdotally revealed that vultures appeared to vary in their wariness to humans.. Since tolerance of human activity might also influence tolerance of or perceived risk of the inflatable deterrents we sought to test this by categorizing sites using a measure of vulture response to human presence. We incorporated flight initiation distance (FID), the distance at which vultures would allow a human to approach before initiating flight^32^, as our measure of tolerance for human presence (hereafter human tolerance). This metric has been used with vultures in similar contexts^19^. FID was obtained by having an invidivual move in a straight line toward multiple vulutres (> 2) and noting the distance at which vulures initiated flight. All FID measurments were taken at least one day prior to the start of data collection. We categorized site FID into three categories of site human tolerance; low (FID > 10 m), moderate (FID 5–10 m); high (FID < 5 m) (Table S1).

### Statistical methods

We conducted an a priori power analysis to determine an approximate estimate of the needed sample size to detect a significant difference in vulture abundance at a site due to inflatable deterrent deployment. Since the expected effect is unknown, effect size was estimated based on results from similar avian deterrent studies^23,24^. An effect size of 0.65 was used which is considered a large effect size using Cohen’s^33^ criteria. The projected sample size needed was approximately *n* = 10 for the simplest between group comparisons. Based on these calculations, we aimed to test inflatable deterrent equipment at a minimum of 10 unique sites in the southeastern United States. Furthermore, we attempted to increase our sample size to 20 since the expected effect was unknown and effect size had to be estimated leading to some uncertainty. However, seven sites had to be removed due to lack of consistency with vulture presence (i.e., vultures left site prior to the completion of every phase).

We used the nonparametric Kruskal–Wallis test to determine if vulture abundance differed across inflatable deterrent phases and with distance from inflatable deterrent during the treatment phase. We also used the Kruskal–Wallis test to determine if vulture abundance differed across site human tolerance category (low, moderate, high). To evaluate potential vulture habituation to inflatable deterrent units, we used the nonparametric Wilcoxon Rank Sum test to compare vulture abundance between the first and last days of treatment. We used non-parametric tests due to unequal sample sizes and uneven variance across groups with non-normal distributions. We used post-hoc Dunn’s multiple comparison tests with a Bonferroni correction to determine which groups differed significantly. For all analyses, we combined turkey vulture and black vulture data as 85% of the vultures observed at sites were black vultures and when analyzed separately results did not vary by species.

We used mixed effects models to determine what factors in addition to inflatable deterrents may have influenced vulture abundance at a site each day. For all mixed effect model analyses, we used site as the blocking factor and day as the sampling unit. The average number of vultures present each day was the response variable. We had six competing models to determine if factors including the presence of inflatable deterrents influenced vulture abundance at sites (Table 2). We evaluated weather variables, including wind speed (m/s), temperature (°C), and cloud cover (%) to determine if weather influenced vulture abundance at a site. Weather data were accessed via the National Weather Service (NWS) weather stations and gathered from the nearest weather station to each site. Additionally, we evaluated site attributes such as area (hectares) and site human tolerance level. Models were fit using the R package “glmmTMB”^34^. We used a negative binomial distribution as the Poisson distribution showed substantial overdispersion. We used the R package “DHARMa” to evaluate model fit^35^.

**Table 2:** A priori model hypotheses of site attributes influencing vulture abundance at sites in the southeastern United States, 2021–2023.

Hypothesis	Model
Phase	Y = Phase
Human Tolerance	Y = Tolerance
Human Tolerance*Site interaction	Y + Tolerance*Site
Weather	Y = Temperature + Wind Speed + Cloud Cover
Area	Y = Area
Global	Y = Phase + Tolerance + Temperature + Wind Speed + Cloud Cover + Area
Null	Y = Site

Y = vulture abundance at a site/day; Phase = pre-treatment, treatment, post-treatment; Site Human Tolerance = low, moderate, high; Area = size of site (hectares). Site was included in all models as a random effect.

We used Akaike’s information criterion corrected for small sample sizes (AIC_C_) to determine which of the a priori models were most parsimonious. We calculated ΔAIC_C_ values and model weights (*w*_*i*_) to determine the distance between the best model and other models in the candidate set. To examine model variability, we also calculated 95% confidence intervals for the parameter estimates. We considered parameter estimates with confidence intervals that did not overlap zero to be useful predictor variables. We included site as a random effect in all models and also included a null model with site as a random effect to determine if vulture abundance was influenced by site alone. All statistical analyses were performed with R 4.1.2^36^.

### Ethical approval

Our methods were reviewed under the USDA National Wildlife Research Center (NWRC) QA-3318 and approved by the NWRC Institutional Animal Care and Use Committee. Our experiment was completed in accordance with the American Veterinary Medical Association guidelines. No animals were injured during this study.

## Results

Black vultures were present at all sites whereas turkey vultures were only present at 9 of 13 (69%) sites. At 7 of the 9 sites where turkey vultures were present, they made up less than 10 of the total number of vultures. However, at 2 of 9 sites, they made up more than 75% of the total number of vultures. Overall, 85% of all vultures observed were black vultures.

**Figure 2:**
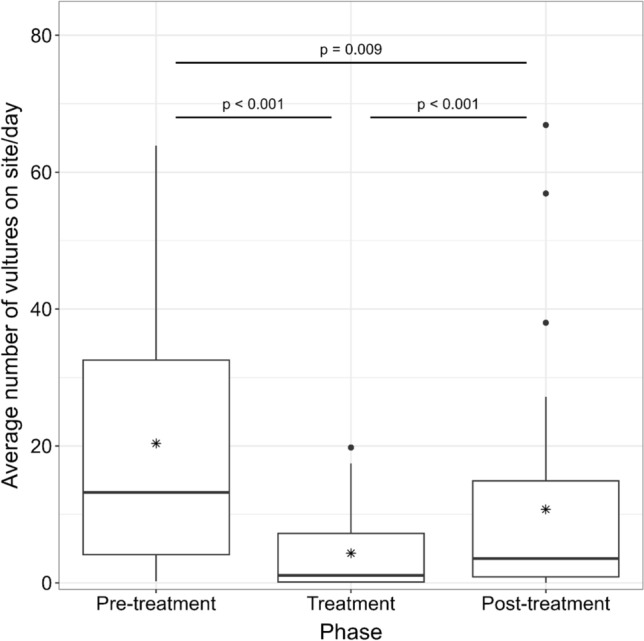
Daily average number of vultures on site across phase (pre-treatment, treatment, post-treatment), southeastern United States, 2021–2023. Within each box plot, the dark central horizontal line represents the median, the asterisk represents the mean, and the closed circles represent outliers. *P* values calculated from Dunn’s multiple comparison test.

Vulture abundance significantly differed across treatment phase (Kruskal–Wallis *χ*^2^ = 272.69, *p* < 0.001; Fig. 2). The number of vultures significantly decreased between pre-treatment and treatment phases (Kruskal–Wallis *χ*^2^ = 272.69, *p* < 0.001) with an 82% reduction in the number of vultures at the site. However, the number of vultures increased during the post-treatment phase (Kruskal–Wallis *χ2* = 272.69, *p* < 0.001) with an overall reduction in the number of vultures between the pre-treatment and post-treatment phases of 48%.

Similar patterns were observed when categorizing vultures by age (i.e., hatch-year, after-hatch-year). Both after-hatch-year (Kruskal–Wallis *χ*^2^ = 223.31, *p* < 0.001) and hatch-year birds (Kruskal–Wallis *χ*^2^ = 181.83, *p* < 0.001) significantly differed across treatment phase (Supplemental materials: Fig. S1).

**Figure 3:**
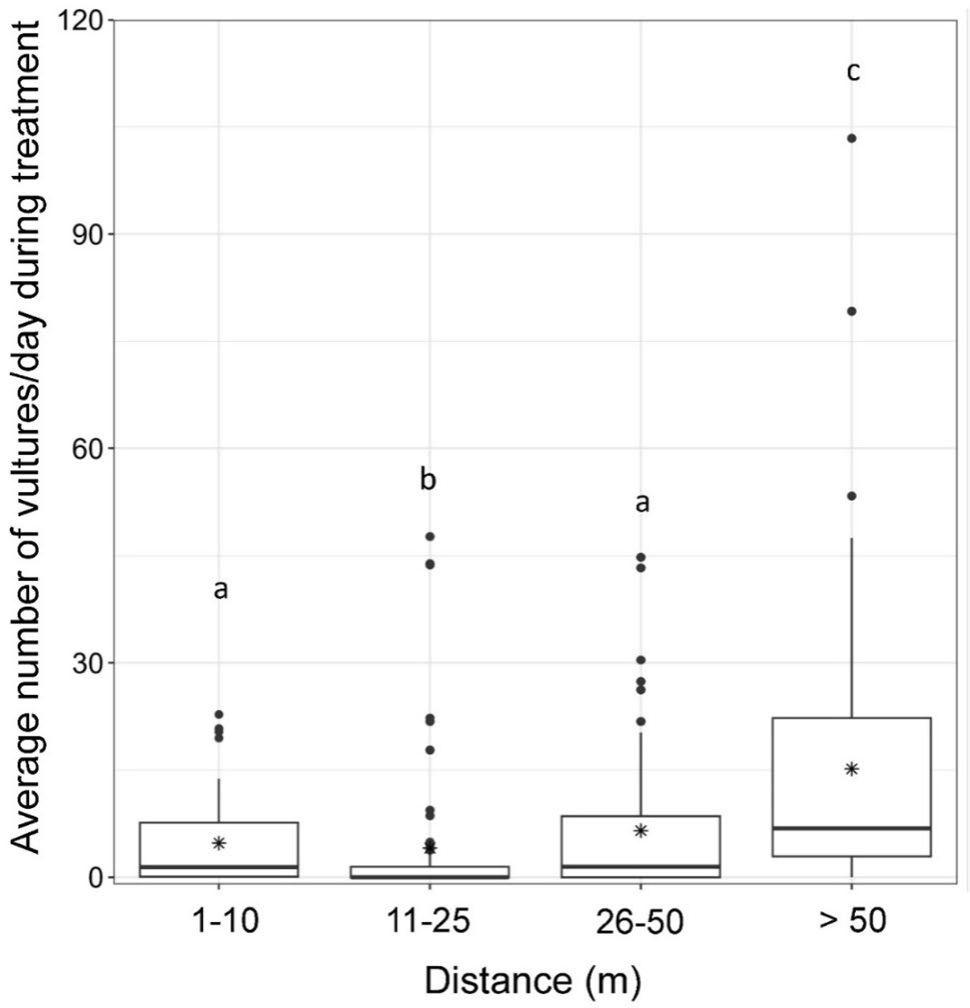
Daily average number of vultures during treatment phase, southeastern United States, 2021–2023. Distance (m) is distance from inflatable deterrent units. Within each box plot, the dark central horizontal line represents the median, the asterisk represents the mean, and the closed circles represent outliers. Letters indicate significant differences (α = 0.05) using Dunn’s multiple comparison test.

During the treatment phase, vulture abundance significantly differed with distance from inflatable deterrents (Kruskal–Wallis *χ*^2^ = 467.66, *p* < 0.001; Fig. 3). Vulture abundance was similar from 1 to 50 m from inflatable deterrent (Fig. 3). However, vulture abundance significantly increased > 50 m from inflatable deterrents when compared to vulture abundance near inflatable deterrents (Fig. 3).

**Figure 4:**
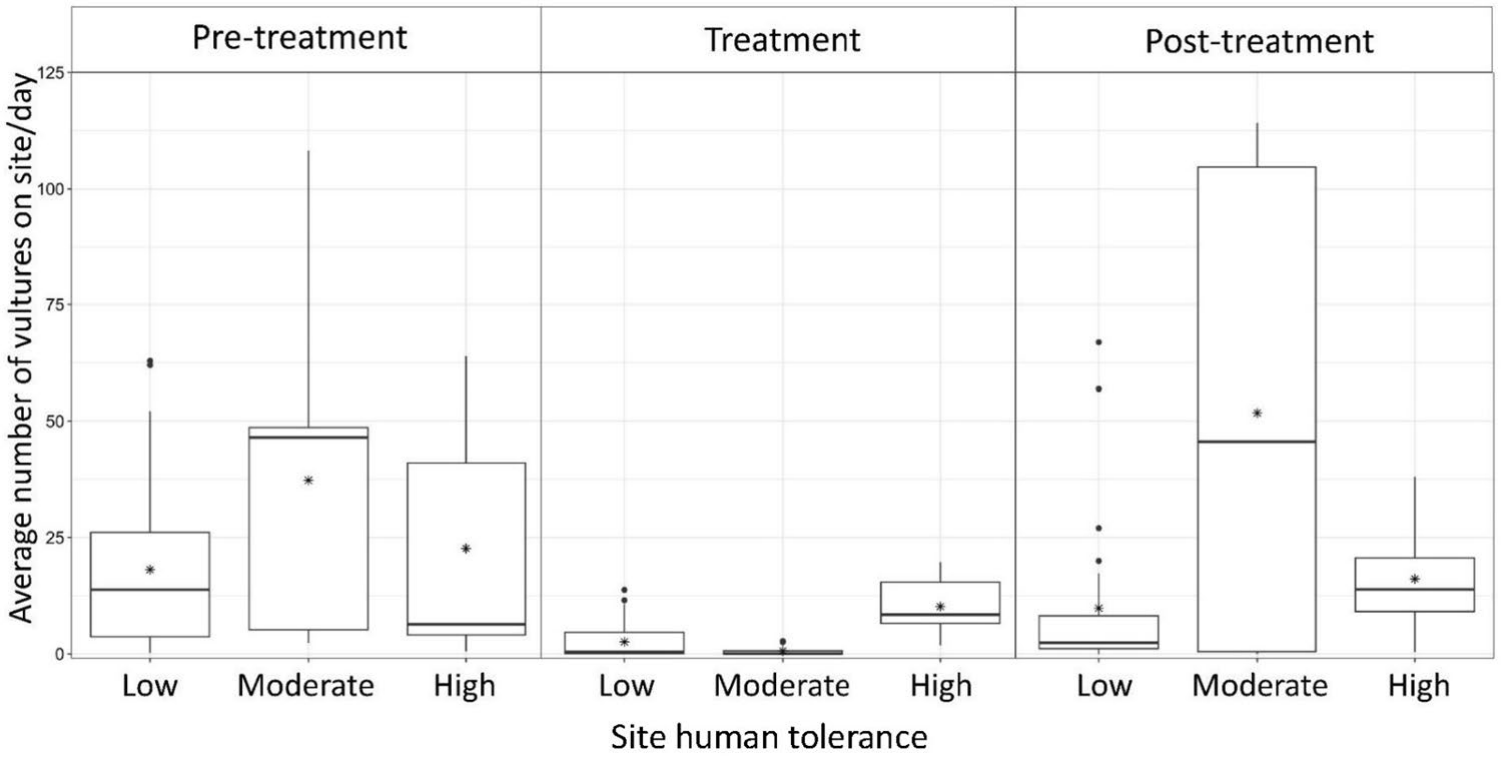
Daily average number of vultures on site across phase (pre-treatment, treatment, post-treatment) and site human tolerance level (low (*n* = 2), moderate (*n* = 8), high (*n* = 3)), southeastern United States, 2021–2023. Within each box plot, the dark central horizontal line represents the median, the asterisk represents the mean, and the closed circles represent outliers.

The effectiveness of inflatable deterrents differed based on treatment phase and site human tolerance level (Kruskal–Wallis *χ*^2^ = 545.93, *p* < 0.001; Fig. 4). The greatest reduction in vulture abundance was between pre-treatment and treatment phases at sites with low (n = 8) and moderate (n = 2) levels of site human tolerance with an 86% and 98% reduction, respectively (Fig. 4). Vulture abundance at high human tolerance sites (n = 3) dropped 55.10% between pre-treatment and treatment phases. The average number of vultures increased between the treatment and post-treatment phases, with the greatest increase observed at sites with low and moderate levels of site human tolerance (Fig. 4).

Overall there was no significant difference in vulture abundance between the first and final days of treatment across all sites (*W* = 84, *p* = 0.977). Furthermore, at 85% of sites there was either no observed change or a reduction in vulture abundance between the first and final days of treatment.

The treatment phase model was the top model (*w*_*i*_ = 0.99, marginal R^2^ = 0.22, conditional R^2^ = 0.76; Table 3) for explaining vulture abundance at sites. The phase model contained the variables for inflatable deterrent phases, pre-treatment, treatment, and post-treatment. The model accounted for 99% of the Akaike weight. The pre-treatment phase had the highest vulture abundance whereas the treatment phase had the lowest vulture abundance (Table 4). All other models were > 4 ΔAIC_C_ away from the top model.

**Table 3:** Results of generalized mixed models for vulture abundance at sites in the southeastern United States, 2021–2023.

Model	*k*	AIC_c_	∆AIC_c_	*w*_i_	R^2^ marginal	R^2^ conditional
Phase	2	922.41	0.00	0.99	0.22	0.76
	:	:	:	:	:	:
Null	1	979.87	57.46	0.00	0.00	0.62

Only models with ∆AICc < 4 and null models are shown and considered plausible. Model are described with number of parameters (k), AICc values, differences in AICc values between the best model and each candidate model (∆AICc), AICc weights (*w*_*i*_), the marginal R^2^ (incorporates variance explained by fixed factors), and conditional R^2^ (incorporates variance explained by both fixed and random factors).

**Table 4:** Top model parameter estimates (β) and confidence intervals (LCL, UCL) for the phase model explaining vulture abundance at sites in the southeastern United States, 2021–2023.

Parameter	β	LCL	UCL
Intercept	2.26	1.65	2.87
Phase			
Pre-treatment	0.50	0.14	0.86
Treatment	− 1.10	− 1.42	− 0.70
Post-treatment	0		

## Discussion

We conducted a multi-site investigation across the southeastern United States, a region of the country experiencing increasing human-vulture conflict^9^. Our most germane finding was that inflatable deterrents substantially reduced vulture abundance while the tool was deployed (Fig. 3). Compared to the pre-treatment phase, overall vulture abundance reduction during the twenty-one-day post-treatment phase was only 48% as compared to 82% for the seventeen-day treatment phase. Thus, site managers should expect local vulture abundance to increase once deterrents are inactivated or removed. Furthermore, we observed an increase in vulture abundance with increased distance (> 50 m) from the inflatable deterrent tool. This finding suggests that inflatable deterrents may not be as effective at larger sites with widespread vulture damage issues. More information is needed to determine if the deployment of more inflatable deterrent device units or the combined use of inflatable deterrents and other management strategies (i.e., pyrotechnics) may discourage widespread vulture damage at these sites.

Study site location did not appear to influence tool effectiveness (Table 3). The majority of historical and contemporary investigations focused on testing effectiveness of deterrents for vultures have employed study designs containing only a single or sparse number of study sites^13,19,25,26,37^, and this has been identified as an area of concern^9^. However, our results suggest that ascertaining tool effectiveness using only a few study sites may be appropriate. However, a robust multiple study site design such as the one we employed is likely more germane and appropriate from an inference-to-a-larger-population perspective. Whereas we did not observe differences in tool effectiveness at the site level, we did observe differences in the effectiveness of the inflatable deterrent based on vulture site human tolerance. A > 85% reduction in vulture abundance was observed at sites classified as exhibiting low and moderate vulture site human tolerance, inflatable deterrents were less effective at high human tolerance sites, at a 48% reduction. Frightening devices, such as inflatable deterrents, are most effective in situations where animals exhibit neophobia^38^. It is possible that sites categorized as high human tolerance were also sites where vultures are routinely subjected to human disturbance and new objects, thereby resulting in inflatable scarecrows being less effective as a vulture tool.

Our study has several limitations that are worth noting and may be improved upon. First, though our study design included a treatment and post-treatment phase, those spanned only two and three weeks, respectively. As a result, our study did not test for vultures reaction to long-term deployment or long-term absence, of the deterrent. This was by design, as we felt that including a robust number of sites across the southeastern United States was a sounder study design than concentrating limited resources on select sites for longer periods. Future investigation should investigate longer term effectiveness and potential for habituation. For the latter, to speak definitively on habituation and sensitization would require monitoring a marked population^39^, which to our knowledge has not been conducted for vultures within a human-wildlife conflict context. Second, in order to maintain continuity throughout our study, we used the same deterrent activation sequence at all sites (deterrents activated every 5 min for two hours). Future investigation could include variations in the activation sequence, such as different timing for each unit, or using timers with a random timing function. This may also influence long-term effectiveness and potential for habituation. Third, by using a reduction in vulture abundance as a measure of deterrent effectiveness, we assumed that a lesser number of vultures present equated to less damage/less vulture-human conflict. Future investigations could devote more resources to measuring damage in addition to local vulture abundance, despite our approach aligning with previous works on tool testing for vultures^13,19,37^. Future works could include formally surveying wildlife land managers to determine what reduction in vultures at sites would be needed for a vulture deterrent to be considered effective. Last, because birds were not marked, we could not determine the proportion of counted birds that were migratory or not; we feel this did not influence our overall results as turkery vultures were observed far less than black vultures.

Vultures clearly perceived inflatable deterrents as risky and adjusted their behavior accordingly. What remains nebulous is the mechanisms driving this behavior. Black and turkey vultures are rarely predated upon by raptors or mammalian carnivores^4,31^ thus it could be argued that their observed response to deterrents may have been grounded in fear of novelty rather than antipredator behavior (Bronson 1968). Sites where vultures exhibited a lower tolerance to human approach coincided with sites with higher deterrent effectiveness. These more wary of human approach vultures may have perceived humans as riskier due to previous negative experiences, such as being chased, harassed, or witnessing members of their social group being dispatched by humans. Further complicating matters is that the visual deterrent we tested is not intended to resemble a human, as is the case with more traditional human effigy scarecrows^17^. Future research on this topic is clearly warranted. To our knowledge, ours is the first investigation of testing an automated visual deterrent on a raptor species. Our results reveal that the number of vultures present can be drastically reduced at sites with low to moderate levels of human tolerance; less effectiveness can be expected at sites with high human tolerance. The flight initiation distance we employed can be used by practitioners to assess site human tolerance. We recommend the use of multiple inflatable deterrents spaced less than 50 m apart. Our study sites were limited to areas where human-vulture wildlife conflict was tied to property damage and impact to recreation, though since the completion of our study, the deterrent has been used with reported success at several residential properties in Arkansas (Tyler Gregory, USDA APHIS Wildlife Service, personal communication). Caution is warranted when considering applying inflatable deterrents to other types of human-vulture conflict, especially those associated with a large spatial footprint (e.g., large farms with reported black vulture livestock depredations).

### Supplementary Information

Supplementary Information.

## Data Availability

Data are available on Zenodo: 10.5281/zenodo.8386756.
