# Supplementary material for: Efficacy of an inflatable deterrent for reducing New World vulture human-wildlife conflict
Source: Sci Rep. 2024 Mar 19;14:6622. doi: 10.1038/s41598-024-56941-2 (PMC10951350; doi:10.1038/s41598-024-56941-2)
Supplement: Supplementary file 1 — Supplementary Information. [file 41598_2024_56941_MOESM1_ESM.docx]

**SUPPLEMENTAL FIGURES AND TABLES**

**Table S1**. Description of inflatable scarecrow study sites and vulture-related damage at the site in the southeastern United States, 2021-2023

| Site | Site description | Vulture damage | Human Tolerance | Area  (hectares) | Date |
| --- | --- | --- | --- | --- | --- |
| **ALABAMA** |  |  |  |  |  |
| Wheeler dam | Hydroelectric dam and associated parking lots and boat ramp located on Tennessee River | Vulture fecal matter and feathers create unsafe conditions on dam and risk of power outages. Vultures damage vehicles in nearby parking lots. | Low | 0.8 | 27-July-2022 to  30-August-2022 |
| Wilson dam | Hydroelectric dam and associated parking lots located on Tennessee River | Vulture fecal matter and feathers create unsafe conditions on dam and risk of power outages. Vultures damage vehicles in nearby parking lots. | Low | 0.10 | 2-August-2022 to  30-August-2022 |
| **ARKANSAS** |  |  |  |  |  |
| Bull Shoals dam | Hydroelectric dam located on the White River | Vulture fecal matter and feathers create unsafe conditions and risk of power outages. | Low | 0.10 | 29-January-2023 to  23-February-2023 |
| **GEORGIA** |  |  |  |  |  |
| Jekyll Island | Waste management site | Vultures frequent dumpsters and spread trash around area. | Low | 0.10 | 23-January-2023 to  31-March-2023 |
| **FLORIDA** |  |  |  |  |  |
| Blue Springs | Springs recreational area | Vulture fecal matter and feathers create unsafe conditions in picnic areas. Vultures also frequent dumpsters and damage picnic area. | Low | 0.40 | 30-May-2023  5-July-2023 |
| Carlos E. Haile Middle School | Farm animal enclosures | Vultures harass farm animals kept at school, including cows and pigs. | Low | 0.40 | 20-March-2023 to  24-April-2023 |
| East River High School | School track and baseball fields | Vulture fecal matter and feathers created unsafe conditions. Vultures also damage track and baseball equipment. | Low | 0.20 | 23-September-2022 to  29-November-2022 |
| Everglades National Park | Visitor center parking lot | Vultures damage rubber and vinyl parts on visitor vehicles. | High | 0.40 | 18-January-2022 to  24-February-2022 |
| Lake Wauburg | Lakefront beach recreational area | Vulture fecal matter and feathers created unsafe conditions. Vultures damage recreational equipment, such as kayaks, pool furniture covers, etc. | Moderate | 0.10 | 15-September-2021 to  26-October-2021 |
| Salt Springs | Springs recreational area | Vulture fecal matter and feathers created unsafe conditions. Vultures also damage visitor personal belongings. | High | 0.81 | 6-June-2022 to  2-August-2022 |
| Santa Fe Teaching Zoo | Small zoo | Vultures frequent animal enclosures and harass animals. | Moderate | 0.40 | 2-November-2021 to  14-December-2021 |
| Silver Glen Springs | Springs recreational area | Vulture fecal matter and feathers created unsafe conditions. Vultures also damage visitor personal belongings. | High | 0.20 | 6-June-2022 to  27-July-2022 |
| **TENNESSEE** |  |  |  |  |  |
| Douglas dam | Campground and boat ramp located at Douglas dam on the French Broad River | Vulture fecal matter and feathers created unsafe conditions. Vultures damage vehicles at boat ramps and campground. | Low | 0.40 | 22-August-2022 to  13-September-2022 |


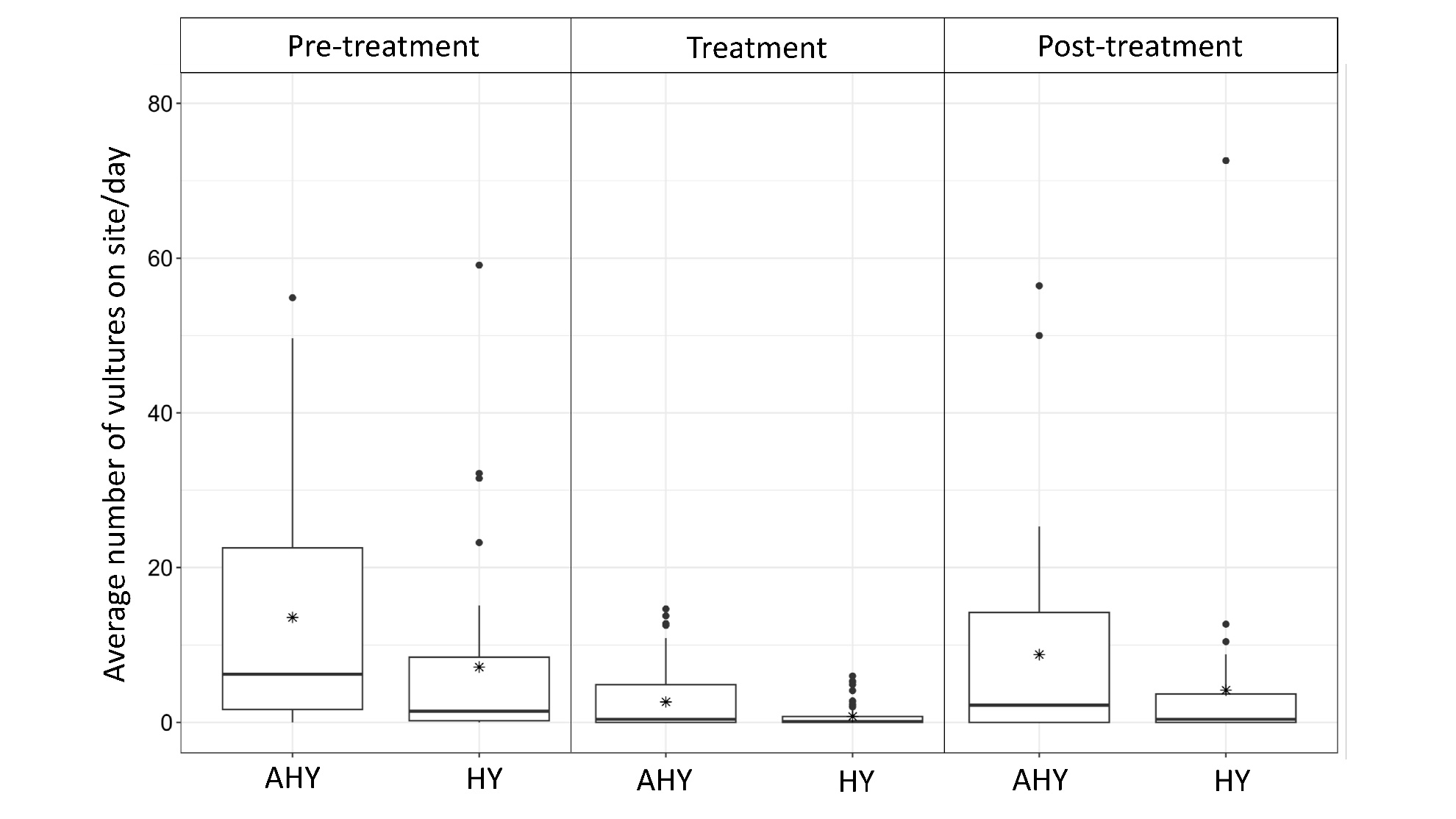


**Fig S1**. Vulture abundance by age (AHY: after-hatch-year, HY: hatch-year) across phases at sites in the southeastern United States, 2021-2023.
